# Assessment of vector competence of UK mosquitoes for Usutu virus of African origin

**DOI:** 10.1186/s13071-018-2959-5

**Published:** 2018-07-03

**Authors:** Luis M. Hernández-Triana, Maria Fernández de Marco, Karen L. Mansfield, Leigh Thorne, Sarah Lumley, Denise Marston, Anthony A. Fooks, Nick Johnson

**Affiliations:** 1 Wildlife Zoonoses and Vector-borne Diseases Research Group, Animal and Plant Health Agency, Woodham Lane, New Haw, Addlestone, Surrey, KT15 3NB UK; 2 Faculty of Health and Medical Science, University of Surrey, Guildford, Surrey, GU2 XH UK; 3 Public Health England, Porton Down, Salisbury, SP4 0JG UK; 4 Department of Clinical Infection, Microbiology and Immunology, Institute of Infection and Global Health, University of Liverpool, Liverpool, UK

**Keywords:** *Culex pipiens*, Usutu virus, Vector competence, UK, Mosquito

## Abstract

**Background:**

Usutu virus (USUV) is an emerging zoonotic virus originally from sub-Saharan Africa. It has been introduced into Europe on multiple occasions, causing substantial mortality within the Eurasian blackbird (*Turdus merula*) population. It is transmitted by the mosquito species *Culex pipiens* in Europe and Africa. Vector competence studies indicate that European strains of USUV are readily transmitted by indigenous *Cx. pipiens*. However, there is limited information on the ability of an African strain to infect European mosquitoes.

**Methods:**

We evaluated the ability of African strain SAAR-1776 to infect two lines of *Cx. pipiens* colonised within the United Kingdom (UK). Mosquitoes were fed blood meals containing this virus and maintained at 25 °C for up to 21 days. Individual mosquitoes were tested for the presence of virus in the body, legs and an expectorate saliva sample. Changes to the consensus of the virus genome were monitored in samples derived from infected mosquitoes using amplicon based next generation sequencing.

**Results:**

Infection, dissemination and the presence of virus in saliva in one mosquito line was observed, but no evidence for dissemination in the second mosquito line. This suggests a strong barrier to infection in UK *Cx. pipiens* for this strain of USUV. When comparing the genome of input virus within the blood meal with USUV recovered from an infected mosquito, we observed limited changes in the consensus genome sequence.

**Conclusions:**

The evaluation of vector competence of UK populations of *Cx. pipiens* for Usutu virus suggests a limited susceptibility to infection with USUV strain SAAR-1776 of African origin. However, within a single mosquito there was complete dissemination and expectoration of USUV, indicating that infection, and potentially transmission, is possible. Sequence changes were observed that may represent early adaption to the mosquito host and could reflect the early events of USUV establishment in European mosquito populations.

**Electronic supplementary material:**

The online version of this article (10.1186/s13071-018-2959-5) contains supplementary material, which is available to authorized users.

## Background

Usutu virus (USUV) is an emerging, mosquito-borne flavivirus that belongs to the Japanese encephalitis virus (JEV) antigenic complex [1, 2]. Like the closely related West Nile virus (WNV), USUV is maintained and transmitted primarily by members of the mosquito genus *Culex* with birds acting as amplifying hosts. Human infection is common [3–6], but disease is rarely reported, usually associated with immunocompromised individuals [7]. The virus was first isolated in 1959 from *Culex neavei* collected near the Usutu River, Natal, South Africa [8]. Subsequently, it has been recorded in birds and other mosquito species including *Cx. perfuscus*, *Cx. univitattus* and *Cx. quinquefasciatus*, throughout sub-Saharan countries [9]. Migratory birds are considered responsible for short and long-distance dispersal of USUV [9]. The first documented evidence of USUV introduction to Europe was during a retrospective study of dead Eurasian blackbirds (*Turdus merula*) in 1996, in Italy [10]. It was next recorded in Austria in 2001, where it again caused significant mortality among Eurasian blackbirds [11]. The virus appeared to overwinter causing further deaths amongst avian species. Since then, USUV has spread to many European countries including the Czech Republic, Germany, Hungary, Spain and Switzerland [2, 9, 12]. In 2016, the virus was recorded in the Netherlands causing widespread die-offs of blackbirds and captive great grey owls (*Strix nebulosa*) [13].

Surveillance of European mosquito species [14] has detected USUV predominantly in the northern house mosquito, *Culex pipiens* (*s.l.*), one of the most abundant species in the northern hemisphere. In addition, USUV has been isolated from *Cx. modestus* [15] and *Aedes albopictus* [16]. Phylogenetic studies indicated there have been multiple introductions with onward transmission of USUV in Europe, and that these viruses are distinct from those circulating in Africa [9]. Genetic variation is indicative of local adaptation of USUV to European populations of *Cx. pipiens* following its introduction. The vector competence of *Cx. pipiens* (*s.l.*) colonised in the Netherlands has been investigated and demonstrated that this species is highly susceptible to infection with a European-derived strain of USUV [17]. In this study, 69% of infected mosquitoes could expectorate USUV in saliva suggesting that they could efficiently transmit virus. However, it is not clear that an African strain is equally infectious in European *Cx. pipiens* mosquitoes. This would be an early event in the introduction of African mosquito-borne virus in Europe, but one that has presumably occurred on multiple occasions.

In the United Kingdom there has been no evidence for autochthonous vector transmission of a mosquito-borne arbovirus since the 19th century [18], although Buckley et al. [19] reported seropositivity in sentinel chickens for West Nile virus, Usutu virus, and Sindbis virus in the UK. Targeted surveillance in locations where migratory birds and mosquitoes are abundant has found no evidence for the presence of arboviruses [20]. However, recent studies have shown that under experimental conditions, UK-derived *Aedes* (*Ochlerotatus*) *detritus* is a potential vector for JEV at 23 °C and 28 °C [21] and WNV [22]. We assessed the ability of an African strain of USUV to infect colonised strains of *Cx. pipiens* derived from the UK. In addition, we monitored the genomic sequence of the infecting virus to evaluate virus adaptation.

## Methods

### Colonization of mosquitoes

*Culex pipiens* species has two ecological forms, *Cx. pipiens* form *pipiens*, and *Cx. pipiens* form *molestus*, which are morphologically indistinguishable. They are found sympatrically and can hybridize along their distribution range [23], with hybrids said to have a greater vector competence for certain arboviruses [24, 25]. Both forms and a hybrid are present in the UK. Two colonised lines: a *Cx. pipiens* typical form (Caldbeck: CBK) and *Cx. pipiens* hybrid form (Brookwood: BKW) were obtained from The Pirbright Institute [26]. Details of mosquito maintenance are provided in Additional file 1: Text S1.

### Cells and viruses

The USUV strain SAAR-1776 originally isolated from *Cx. neavi* in South Africa (provided by Professor E. Gould, Centre for Hydrology and Ecology), was passaged three times in Vero C1008 cells to a titre of 4.0 × 10^6^ PFU/ml. Vero cells were maintained in Dulbecco Modified Eagle’s Medium (DMEM) (Sigma-Aldrich, United Kingdom) containing 10% heat-inactivated Foetal Calf Serum (FCS), 2 mM L-glutamine and 50 μg/ml Penicillin/Streptomycin (Sigma-Aldrich).

### Assessment of vector competence of UK *Cx. pipiens* mosquitoes

Both UK lines of *Culex pipiens* (CBK and BKW) were tested for their vector competence for the SAAR-1776 strain of USUV at 25°C. Mosquitoes were provided an infectious blood meal composed of defibrinated horse blood, adenosine 5’-triphosphate (final concentration 0.02 mM) and virus stock to give a final virus concentration of 1.0 × 10^6^ PFU/ml using a Hemotek membrane feeding system (Hemotek Ltd Accrington, Lancashire, UK). Five to ten day-old adult female mosquitoes were allowed to feed for a minimum of 16 h at room temperature. Blood-fed and non-blood-fed specimens were anaesthetized with Triethylamine (TEA) FlyNap® (Blades Biological Limited, Edenbridge, UK) and separated in groups of 10–20 mosquitoes, which were placed in microhabitat pots of 118 × 73 mm in dimension (www.bugzarre.co.uk). Once fully recovered, the mosquitoes were maintained at 25 °C at a relative humidity of between 55–65% with a light cycle of 12:12 light:dark cycle. At 0, 7, 14 and 21 days post-infection (dpi) mosquito groups were processed to provide body, legs, head and saliva samples. Further details of mosquito infections are provided in Additional file 2: Text S2.

To estimate vector competence, we used previous methods [27–30]. Infection rate were calculated by the number of USUV positive mosquitoes with an infected body per number tested at each time point. Dissemination efficiency was calculated as the number of USUV positive mosquitoes with an infected leg per number tested at each point. Transmission efficiency was calculated as the number of mosquitoes with USUV detected in their saliva per number of mosquitoes with an infected leg at each time point.

### Processing of samples for reverse transcription (RT) PCR

Legs/wings, head, and body (thorax/abdomen) of female *Cx. pipiens* were homogenized individually in flat-cap homogenization tubes (Qiagen, Mancherster, United Kingdom a) containing 300 μl of EMEM media containing 10% foetal bovine serum (FBS), penicillin/streptomycin 1% and amphotericin-B 1% and one 3 mm stainless steel bead; beads were not added to the tubes containing the saliva. Homogenisation was undertaken using a Qiagen Tissue Lyser (model Retsch MM301) at 25 MHz for 3 min. All tubes were centrifuged for 5 min at 14,000× *rpm* and 30 μl of supernatant was collected for virus isolation assays. The remainder of the homogenate was used for RNA extraction using TriZol (see www.tools.thermofisher.com). The RNA pellet was eluted in 20 μl of nuclease-free water.

USUV RNA was detected using the primers and probe targeting a 91 base pair region of the NS1 as previously published [31]. The real time reverse transcription PCR was performed on the MxPro 3005P thermal cycler (Stratagene,Thermofisher, United Kingdom) in a 25 μl reaction containing: RNase-free water (5.25 μl); 2× QuantiTect RT-PCR Master mix (12.50 μl); Jöst USUV Primer mix (10 μM primer, 1.25 μM probe) (2 μl); QuantiTect RT mix (Qiagen) (0.25 μl), and 5 μl of RNA template. The conditions were as follows: reverse transcription 50 °C for 30 min; reverse transcriptase inactivation 95 °C for 15 min; and PCR amplification and detection 50 cycles consisting of 95 °C for 15 s, 55 °C for 30 s, and final extension 72 °C for 30 s.

### Virus isolation and titration

Plaque assays were performed on those samples positive by PCR. A ten-fold dilution series of supernatant of the homogenized body parts or salivary secretions (30 μl) was prepared, and dispensed onto a confluent monolayer of Vero cells on a 12-well plate (Costar®, Corning Life Sciences, Massachusetts, , USA). The plates were incubated at 37 °C in an atmosphere of 5% CO_2_ for 3 h. After incubation, 1–1.5 ml overlay of warmed 3% carboxymethylcellulose (CMC) in 250 ml 2× EMEM mix (Deionised water, 7.5% sodium bicarbonate, HEPES 1M, L-glutamine 200 mM, FBS, antibiotics (penicillin and streptomycin), and 10× MEME-E) were added to each well. After 7 days incubation, 1 ml of 10% neutral buffered formalin solution was added to each well and the plates left for least 3 h to complete virus inactivation. Wells were stained with 200 μl of 2.3% crystal violet solution.

### Generation of USUV genome consensus sequence

Multiplex PCR that generates amplicons spanning the whole genome in two reactions was achieved following the methodology of Quick and co-workers [32]. A set of 35 oligonucleotide primer pairs were designed using a multiple alignment of European and African USUV sequences (Additional file 3: Table S1) using the Primal Scheme design tool [see 33 for details]. Two reaction tubes were prepared for each cDNA (one for primer Pool A and the other for primer Pool B), and the reactions carried out in a final volume of 25.5 μl [32]: 5 μl of Q5 Reaction buffer (5×,), 0.5 μl of dNTPs (10mM), 0.25 μl of Q5 DNA Polymerase (BioLabs, Massachusetts, USA), 1 μl of respective primer Pool at 10 μM (Primer Pool A or primer Pool B), 2.5 μl of cDNA and 16.25 μl of nuclease-free water. The conditions used were: 45 cycles of 95 °C for 30 s, 95 °C for 15 s and 65 °C for 5 min.

Amplified products were sequenced using the MiSeq (Illumina, San Diego, CA, USA) sequencing platform at the Animal and Plant Health Sequencing Unit. After excluding primer regions using Trimmomatic (see [33] for details), reads were mapped employing an iterative process described previously [33] using a complete USUV genome as the reference sequence (KC754958).

### Statistical analysis

Survival curves for each group of specimens with infected or uninfected blood meals were compared using Kaplan Meier curve analysis and groups compared by Log-Rank test using Graphpad Prism 5 software.

## Results

### Vector competence of colonised UK mosquitoes for USUV

**Fig. 1:**
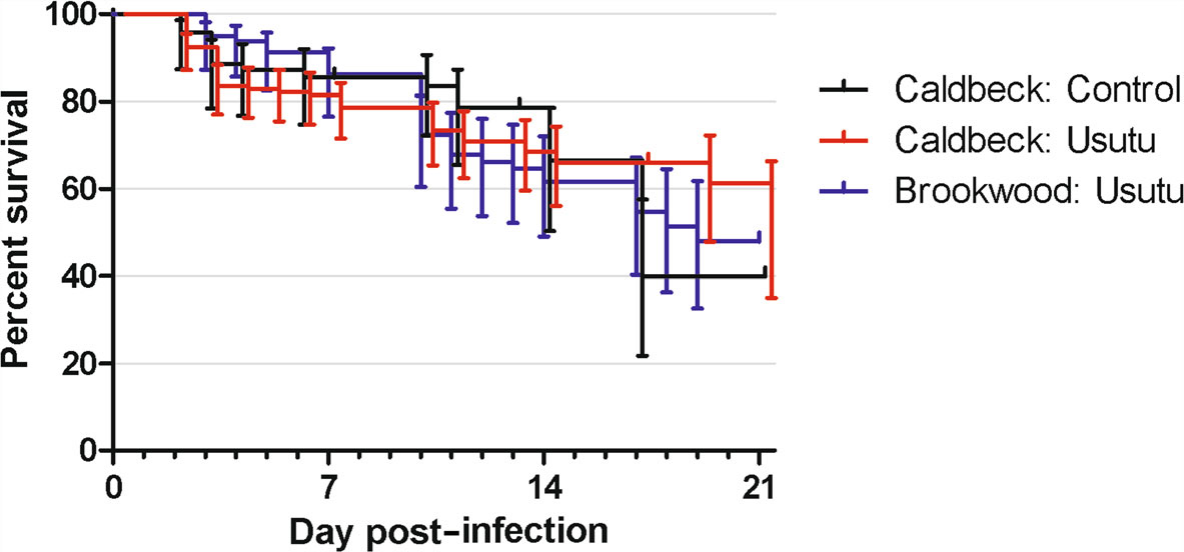
Comparison of survival curves for UK Caldbeck and Brookwood Lines of female *Cx. pipiens* (*s.l.*) infected with USUV, *versus* uninfected females following immobilization with FlyNap

Two *Cx. pipiens* lines were used to investigate the vector competence of UK mosquitoes for an African strain of USUV, both colonised from natural populations. A total of 120 female *Cx. pipiens* typical form (CBK) and 100 female *Cx. pipiens* hybrid (BKW) were offered an infectious blood meal. In addition, 70 females for the CBK were used as non-infected controls. Examination of specimens showed that over 95% took a full blood meal. The exposure of engorged specimens to FlyNap for immobilization did not have a deleterious effect for their recovery as shown at dpi 0 and 3. However, a slightly higher mortality was seen after dpi 14 (Fig. 1). Survival of CBK did not differ significantly from uninfected controls (*P* = 0.0874) or BKW (*P* = 0.9962) (log-rank test).

In both mosquito lines, USUV was detected by PCR in the blood meal (abdomen) at dpi 0. However, the infection rates and transmission efficiency were low, when examined at later time points. Only one CBK specimen had detectable USUV RNA in the body at dpi 14 and one at dpi 21 (Infection rate: 5 % and 14.2%, respectively), while a single specimen from BKW was positive at dpi 21 (Infection rate: 5.5 %) (Table 1). Of the two mosquitoes susceptible to infection in this study, only one specimen in the CBK line had USUV present in the saliva at dpi 14 (100%). No USUV RNA was detected in the saliva in the BKW line.

**Table 1:** Infection (Body), dissemination (Legs) and transmission (Saliva) rates of UK Caldbeck and Brookwood lines of *Cx. pipiens* infected with Usutu virus at 25 °C

	Dpi	*Culex pipiens* (Caldbeck line)	*Culex pipiens* (Brookwood line)
Body	7	0/20 (0%)	0/14 (0%)
	14	1/20 (5%)	0/18 (0%)
	21	1/7 (14.2%)	1/18 (5.5%)
Legs	7	3/20	0/14 (0%)
	14	1/20	0/18 (0%)
	21	0/6 (0%)	0/10 (0%)
Saliva	7	0/20 (0%)	0/14 (0%)
	14	1/1 (100%)	0/18 (0%)
	21	0/1 (0%)	0/18 (0%)

Although the titre of the frozen virus was estimated to be 4 × 10^6^ PFU/ml, and the freshly harvested viral stock (working stock) was originally estimated at 6.3 × 10^6^ PFU/ml, the assessment of virus in the blood meal before and after the blood-feeding yielded a titre of 2 × 10^4^ PFU/ml, equating to a 2-log drop in viral titre. Infectious viral titres (in PFU/ml) in the CBK specimen with positive saliva were: saliva 4.0 × 10^1^, leg/wings 8.3 × 10^3^, head 8.0 × 10^4^, and abdomen 6.8 × 10^5^. All other USUV RNA positive samples detected by RT PCR did not induce cytopathic effect to the Vero cell monolayer.

### Impact of replication in *Cx. pipiens* on the USUV genome of African origin

**Fig. 2:**
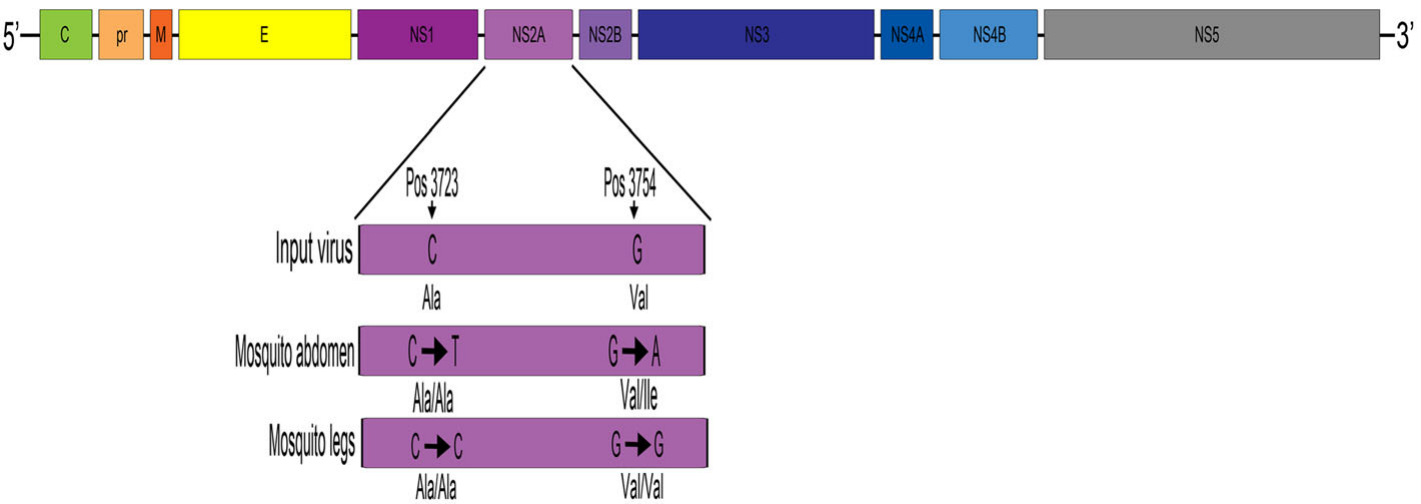
Amino acid substitutions in the polyprotein NS2A of USUV strain SAAR1776 in the abdomen and legs of UK CBK line of *Cx. pipiens* at day post-infection 14

USUV sequence was obtained covering > 97% of the genome from the input virus (10,758 nucleotides) as well as the abdomen (10,758) of the USUV positive CBK mosquito at day 14 post-infection (see Table 2). Two nucleotide substitutions, C3723T and G3754A, were detected in the non-structural protein 2A (NS2A) gene retrieved from the abdomen of the mosquito (Fig. 2, Table 2). These substitutions at consensus level between the input virus and the virus recovered from the mosquito were only observed in the body sample. The nucleation variation at each position is as follows: 3723 - T (99.83%), C (0.08%), G (0.07%), A (0.01%); 3754 - A (99.72%), C (0.12%), G (0.09%), T (0.07%). The USUV recovered from the leg was identical to the input virus albeit with a low depth coverage (Table 2). Despite numerous attempts, no amplicons were obtained from the saliva sample for comparison. These nucleotides changes led to one synonymous amino acid change (A1220A) and one non-synonymous amino acid change (V1209I).

**Table 2:** Consensus sequence changes detected in the abdomen from one *Culex pipiens* CBK mosquito found positive for USUV at day 14

Genome position^a^	Region	Input codon	Depth coverage	Abdomen codon	Depth coverage	Leg codon	Depth coverage	AA change
3723	NS2a	CGC	9232	CGT	10,708	CGC	22	A1220A
3754	NS2a	CGG	9448	CGA	10,862	CGG	24	V1209I

^a^Relative to accession KC754958

*Abbreviations*: *NS2a* non-structural protein, *AA* amino acid

## Discussion

A range of factors have heightened the need to investigate the capacity of UK mosquito species to act as vectors of arboviruses. These include: the detection of virus specific neutralizing antibodies to USUV, WNV and Sindbis virus (SINV) in birds in the UK [19, 34], the recent emergence and spread of exotic mosquito-borne viruses in Europe [35], and the ongoing expansion of invasive species such as *Ae. aegypti*, *Ae. albopictus* and *Ae. japonicus* [35–37]. USUV has repeatedly been introduced into Europe since the 1990s [9, 38]. All USUVs isolated and characterised in Europe are grouped into three lineages based on their geographical location, Europe 1, 2 and 3, and are distinct from viruses isolated in Africa. This suggests that there has been USUV genome evolution following its introduction into European mosquito populations. The co-circulation of WNV and USUV in *Culex* species in Europe have been demonstrated [15, 16, 39], and the vector competence of European population of *Cx. pipiens* for USUV has recently been assessed [17]. The authors concluded that *Cx. pipiens* of European origin is highly competent for both viruses, with a significant increase in vector competence for USUV at higher temperatures. In order to assess the ability of USUV of African origin, we have attempted infection of two lines of UK *Cx. pipiens* with strain SAAR-1776, isolated in South Africa. This is also the first study to investigate the vector competence of UK populations of *Cx. pipiens* for USUV.

In contrast to high infection/transmission rates from experiments with a European USUV isolate, our results showed that only a single specimen of UK CBK *Cx. pipiens* form *pipiens* was positive for USUV RNA in the saliva 14 days after taking a blood meal. A range of factors could influence the competency of a particular mosquito to vector a virus. These include the genetic variability of the virus strains, virus titres used during oral infection, the chosen temperature and selective pressures during laboratory colonization that may change susceptibility to infection [23, 40–42]. This study was conducted with these factors in mind, therefore temperature, viral titre and procedures were all carefully selected to minimise these effects.

The effect of two nucleotide substitutions in the non-structural protein NS2A on the phenotype of the progeny virus is unknown, although it is interesting to note that a change with a deep coverage was only observed in the mosquitoes’ abdomen, where bottlenecks and selective pressures are said to occur while the virus overcome the midgut barrier (Table 2) [43, 44]. The nucleotide substitutions detected in the leg cannot be fully explained because of the low depth retrieved from the analysis (Table 2). Engel et al. [9] proposed that migratory birds can act as potential long-distance dispersal vehicles, the African lineages of USUV have been driven by extensive gene flow, and that the European lineages have been shaped by *in situ* evolution with host-specific mutations also being detected. Their observed mutations at the amino acid level in the NS2A protein (V91A) was suggested to be involved in the formation of the USUV European lineages 1 to 3. Nonetheless, it was concluded that the pathogenicity of specific USUV lineages has been poorly studied and more work is needed to determine the biological characteristics in each lineage.

The previous study investigating USUV infection in mosquitoes infected mosquitoes with the Bologna 09 strain at 4 × 10^7^ PFU/ml and experimentally fed the females for 1 h. Here, we used the USUV isolate SAAR-1776 from South Africa at a final titre of 1.0 × 10^6^ PFU/ml. Furthermore, preliminary infection trials resulted in low blood-feeding success; therefore mosquitoes were fed overnight to increase the number of engorged females. Overnight feeding resulted in large numbers of blood-fed females, and virus isolation confirmed that USUV was still viable in the blood at the end of the feeding period. However, a 2-log drop in virus concentration was observed in the infectious blood meal post-feeding, likely due to the longer feeding period. Numerous authors have proposed there is a clear relationship between virus titer in the blood sample and the infection rate [40] and the low vector competency observed in this study could be related to lower levels of virus in the blood meal, particularly if mosquitoes took a blood meal towards the end of the feeding period. The range of viraemias detected in USUV infected birds is not known, but high, medium and low titres in experimentally infected wild birds with closely related WNV are > 10^6^, 10^4^, 10^6^ and ≤ 10^4^ PFU/ml, suggesting that the titer used in our study was appropriate [45].

## Conclusions

In this study, two UK strains of *Cx. pipiens* challenged with USUV showed reduced competency for an African strain of USUV, although infection, dissemination and virus expectoration can occur. As a result, further vector competence experiments employing higher titres of the USUV at temperatures representative of the UK climate, or using recently isolated European USUV strain, are required to test the competency of indigenous mosquitoes for mosquito-borne viruses.

## Additional files

Additional file 1: **Text S1.** Mosquito maintenance. (DOCX 15 kb)

Additional file 2: **Text S2.** Membrane-based infection studies in mosquitoes. (DOCX 17 kb)

Additional file 3: **Table S1.** Primers used to amplify USUV genomes. (DOCX 22 kb)

## Abbreviations

BKW: Brookwood
CBK: Caldbeck
JEV: Japanese encephalitis virus
SIV: Sindbis virus
USUV: Usutu virus
WNV: West Nile virus
