# Supplementary material for: Assessment of vector competence of UK mosquitoes for Usutu virus of African origin
Source: Parasit Vectors. 2018 Jul 3;11:381. doi: 10.1186/s13071-018-2959-5 (PMC6029037; doi:10.1186/s13071-018-2959-5)
Supplement: Supplementary file 1 — Text S1. Mosquito maintenance. (DOCX 15 kb) [file 13071_2018_2959_MOESM1_ESM.docx]

**Additional file 1.** **Text S1.** Mosquito maintenance

Specimens from both lines of *Cx. pipiens* used in this study were sourced from the APHA insectary. *Culex pipiens* colony were established at APHA in 2015 from The Pirbright Institute via an MTA (No. AHVLA [543]14042014) and they have been morphologically and molecularly characterized in [26]. The colonies were seeded on 25^th^ January 2015 from 200 adults and numerous eggs rafts. Larvae were reared in plastic trays (15 × 30 × 5 cm) in filtered water and left to pupate and emerge in the same tray. Larvae were fed daily with fish food (Aquarian), and the water was changed depending of cleanness. Adults were blood fed on defibrinated horse blood twice a week using a Hemotek feeding system (Hemotek Ltd, UK). Egg bowls filled with filtered water were placed inside the cages and collected twice a week. The insectary was maintained at 25°C±1°C with a relative humidity of 65-70%±3% and 7:7 light: dark cycle. The adults were provided with cotton embedded with 10% sucrose solution*.*
