# Supplementary material for: Assessment of vector competence of UK mosquitoes for Usutu virus of African origin
Source: Parasit Vectors. 2018 Jul 3;11:381. doi: 10.1186/s13071-018-2959-5 (PMC6029037; doi:10.1186/s13071-018-2959-5)
Supplement: Supplementary file 2 — Text S2. Membrane-based infection studies in mosquitoes. (DOCX 17 kb) [file 13071_2018_2959_MOESM2_ESM.docx]

**Additional file: S2.** Membrane-based infection studies in mosquitoes.

All work with infectious blood meals was undertaken in the Animal and Plant Health Agency ACDP containment level 3 facility within a Class I Microbiology Safety Cabinet. Infectious blood meal in a volume of 2 mL was prepared containing: virus stock and defibrinated horse blood (TCS biosciences) at a 1:3 ration and adenosine 5’-triphospahte (ATP) (Thermo Fisher Scientific) as a phagostimulant to a final concentration of 0.02mM. For the control, virus was substituted with Eagle’s Minimum Essential Medium (EMEM). Five to ten days old, adult females were deprived of sucrose for 24 hours prior to feeding and provided only water. The following day, mosquitoes were deprived from water 4-5 hours before infectious blood feeding. Peroral infection was achieved using a Hemotek membrane feeding system using a Parafilm® M membrane (Hemotek Ltd Accrington, Lancashire, UK). The blood feeding was prepared in the afternoon, and the specimens were left to feed throughout the night at room temperature in the dark. In all cases, 500 µL aliquots of the infectious blood meal were taken before and after the mosquitoes feed and stored at -80**°**C for quantification by virus isolation and PCR.

The following day, all specimens were immobilized by placing the cages inside a plastic bag and exposing them to a small piece of cotton embedded with Triethylamine (TEA) FlyNap® (Blades Biological Limited, UK) [26] for less than 10 min. Once all specimens were anesthetized, the engorged mosquitoes were sorted in groups of 10 specimens, gently placed into microhabitat pots (www.bugzarre.co.uk), and left for 2-3 hours within the MSC I to allow constant ventilation for their recovery. Once some specimens were moving or flying in the pots, a small piece of cotton embedded in 10% sucrose was placed on top of the lids. All pots were placed inside a double-clip lock box and taken outside the cabinet to be placed in an incubator at 25°C. A pot with water was kept in the incubator to maintain a relative humidity of 65-70%.

Mosquitoes were sampled at days 0, 7, 14, and 21 days post infection (dpi). All mosquitoes had their saliva collected before being separated into legs/wings, head, and body (thorax, abdomen). This was also achieved by exposing the target microhabitat pot to FlyNap® for less than 10 min. Once all mosquitoes were immobilized, they were placed on their left side, and both wings clipped with the removal of all legs. All tissues were placed in 300 µL of virus diluent media (EMEM), containing 10% Foetal Bovine Serum (FBS), Antibiotics (AB), Penicillin/Streptomycin 1%, and Antifungal (AF) Amphitherocine-B 1%, and frozen at -80°C for subsequent determination of infection, dissemination and transmission rates.

Salivary secretions were collected by inserting the proboscis into 200 µL pipettes tips containing 20 µL of a mixture of virus diluent medium (10 µL), 50% sucrose (5 µL) and adenosine 5’-triphosphate (ATP, 0.02 µM) (5 µL) for 45 minutes. To stimulate salivation, 5 µL of Pilocarpine 1% (Alfa Aesar, Ward Hill, MA, USA) solution containing Phosphate Buffered Saline (PBS) and 0.1% Tween 80 was applied around thorax and abdomen. Indication of signs of salivation was achieved by direct observation of movements of the maxillary palpi and stylets, bubble formation in the media, and engorgement of the mosquito abdomen [20]. The salivary contents were then released into a tube containing 280 µL of virus diluent medium and frozen at -80°C for subsequent determination of transmission rates.
