# Supplementary material for: Assessment of vector competence of UK mosquitoes for Usutu virus of African origin
Source: Parasit Vectors. 2018 Jul 3;11:381. doi: 10.1186/s13071-018-2959-5 (PMC6029037; doi:10.1186/s13071-018-2959-5)
Supplement: Supplementary file 3 — Table S1. Primers used to amplify USUV genomes. (DOCX 22 kb) [file 13071_2018_2959_MOESM3_ESM.docx]

**Additional file: S3. Table S1**. Primers used to amplify USUV genomes.

| **Reaction Name** | **Primer Sequence – LEFT (5’-3’)** | **Primer Sequence – RIGHT (5’-3’)** |
| --- | --- | --- |
| Usutu virus 1 | CGTGAGATTAACACAGTGCCGG | CTCTCTTCTTTCTTGTGCCCCG |
| Usutu virus 2 | CACGGCAATGAAACACCTGACA | CTCCGCTTCGAATGTCTGGTTC |
| Usutu virus 3 | CCCAGAAGACATTGACTGTTGGT | GCCATGATGGTTATGCAGCTGT |
| Usutu virus 4 | CTTCCTTGAGGGAGTCTCTGGT | AGTTGCCATGAGTGTCAGAGCT |
| Usutu virus 5 | CAACTTCACCTGCTCCCTGAAAG | ACGACTGATTGCTTTGTGGCAT |
| Usutu virus 6 | TTCACCAGCCAGCTCAAATTGG | ACCAATGGGAGTGAGATCGGAA |
| Usutu virus 7 | AACTGCAGTACACGGGATCTGA | TCCTGAAGGCTCCTCCAAAGAC |
| Usutu virus 8 | TAGGTGACACAGCTTGGGACTT | ATATTCCTTTCGCGTGGGCTTG |
| Usutu virus 9 | GTGGGTCGACCGGTACAAATTC | CCAAACCCTGGTGGACATGATG |
| Usutu virus 10 | CCCCGACGTAAAAAGAGCTTGG | AGTGATTGTGACGGTGGTACCT |
| Usutu virus 11 | GTTACAAAGTCCAGAGCCAAGGG | AGTAGAGCTCCCACAATAGCCG |
| Usutu virus 12 | CCTTCTGGTGATGTTTCTGGCC | TGGCAGTGGCATTGAGAATTCC |
| Usutu virus 13 | TGGGGGCAGCATTCTTTCAGAT | ATTCAGCTAGACCACCCACGAT |
| Usutu virus 14 | ATGCAATCCCAACAAAAAGCGG | TGTCCCAAAAGACACCTCCTCTT |
| Usutu virus 15 | CATGACGGCACTAGGATTCGCA | ATGTTTATGGCTGCCTTTCCGG |
| Usutu virus 16 | GGTGGACCATGGAAGTTTGACA | GGCATCTTTGATGATCTGGGGG |
| Usutu virus 17 | GCAACTAACAGTTTTGGACCTGC | AGGGGGAGTTGCAGTCATGAAT |
| Usutu virus 18 | ACTGATCCAGCGAGCATAGCAG | AAGTTCGCACCCATCTCTGAGA |
| Usutu virus 19 | CGCAAGTCTTATGACACGGAGTA | AAGCTTTGTCTCTTTCTGGCCC |
| Usutu virus 20 | TCATTGGACTGAAGCAAAGATCATGT | GGACTTCAAGGAATCCCACTGC |
| Usutu virus 21 | AGATGCCAGGGTCTATTCCGAC | GACGTCAGCCATCCACAAGAAG |
| Usutu virus 22 | CGTTCAGAGGAGAGGCATAGGT | CGTGAGAACTACTGTGCTCCCT |
| Usutu virus 23 | ACATTCCATGAACGCTTTGGCA | ATTTTTCATGATTCCGGCCGCT |
| Usutu virus 24 | CTCCACTATGGCTACATGCTGC | CTTCCTCCTGGTCTTCCTCGTT |
| Usutu virus 25 | GCTGGAGCCTCTATAGCTTGGA | GTGACAAGGTTCCAGCCATAGC |
| Usutu virus 26 | TCAGAGGCTACACGAAAGGAGG | GCTCCACTGACCCAGTACATCT |
| Usutu virus 27 | CAACGGAGGTATGGAGGAGGAT | TTGACCAAGGAGCTTGCTGAAC |
| Usutu virus 28 | GACAAGGACCACCCATATCGGA | CATCCACCATTTCCCAGAACCG |
| Usutu virus 29 | GGTGCCATGTTTGAAGAGCAGA | CCTTGGCCTCGTTGTCAAGATC |
| Usutu virus 30 | ACTCAGGTGGGAAAATGTACGC | GCAATCATCTCCACTCACAGCC |
| Usutu virus 31 | TACGCTGTAAGAACCTGGCTCT | TTGCCATCAGCCTCAGATCTCT |
| Usutu virus 32 | GGAATGTTAGGGACACCGCATG | ATGGCCGCGTAGATGTTTTCAG |
| Usutu virus 33 | GGAAGCGGGAAGACATATGGTG | GCAGCACCGTCTACTCAACTTC |
| Usutu virus 34 | TGTGTAGCTTTACTTAGCATCATTTTAGGA | GAAACAGTTCGCATCACCGTCT |
| Usutu virus 35 | CCTGCCTATTGGAAGCGTTCAG | GATCCTGTGGTCTAGTTCCCCA |
